# Dynamics of necroptosis in kidney ischemia-reperfusion injury

**DOI:** 10.3389/fimmu.2023.1251452

**Published:** 2023-11-02

**Authors:** Aspasia Pefanis, Anjan K. Bongoni, Jennifer L. McRae, Evelyn J. Salvaris, Nella Fisicaro, James M. Murphy, Francesco L. Ierino, Peter J. Cowan

**Affiliations:** ^1^ Immunology Research Centre, St Vincent’s Hospital, Melbourne, VIC, Australia; ^2^ Department of Medicine, The University of Melbourne, Melbourne, VIC, Australia; ^3^ Department of Nephrology, St Vincent’s Hospital, Melbourne, VIC, Australia; ^4^ Walter and Eliza Hall Institute of Medical Research, Parkville, VIC, Australia; ^5^ Department of Medical Biology, The University of Melbourne, Parkville, VIC, Australia; ^6^ Drug Discovery Biology, Monash Institute of Pharmaceutical Sciences, Monash University, Parkville, VIC, Australia

**Keywords:** necroptosis, ischemia-reperfusion, necroinflammation, mlkl, acute kidney injury, chronic kidney disease

## Abstract

Necroptosis, a pathway of regulated necrosis, involves recruitment and activation of RIPK1, RIPK3 and MLKL, leading to cell membrane rupture, cell death and release of intracellular contents causing further injury and inflammation. Necroptosis is believed to play an important role in the pathogenesis of kidney ischemia-reperfusion injury (IRI). However, the dynamics of necroptosis in kidney IRI is poorly understood, in part due to difficulties in detecting phosphorylated MLKL (pMLKL), the executioner of the necroptosis pathway. Here, we investigated the temporal and spatial activation of necroptosis in a mouse model of unilateral warm kidney IRI, using a robust method to stain pMLKL. We identified the period 3-12 hrs after reperfusion as a critical phase for the activation of necroptosis in proximal tubular cells. After 12 hrs, the predominant pattern of pMLKL staining shifted from cytoplasmic to membrane, indicating progression to the terminal phase of necroptotic cell death. *Mlkl*-ko mice exhibited reduced kidney inflammation at 12 hrs and lower serum creatinine and tubular injury at 24 hrs compared to wild-type littermates. Interestingly, we observed increased apoptosis in the injured kidneys of *Mlkl*-ko mice, suggesting a relationship between necroptosis and apoptosis in kidney IRI. Together, our findings confirm the role of necroptosis and necroinflammation in kidney IRI, and identify the first 3 hrs following reperfusion as a potential window for targeted treatments.

## 1 Introduction

Ischemia-reperfusion injury (IRI) is a pathological process that occurs when the blood supply to an organ is temporarily reduced and then restored (1). Cellular damage occurs during both the ischemic period and subsequent reperfusion of the kidney. During ischemia, there is a switch from aerobic to anaerobic metabolism (2), resulting in acidosis (3). Cellular ion transport mechanisms are compromised, with increased intracellular calcium, sodium and water causing cellular oedema (4–6). During reperfusion aerobic metabolism is restored and pH is normalised, but reactive oxygen species (ROS) are generated, damaging functional cellular components and ultimately inducing cell death (7–9). The kidney, a highly vascular and metabolically active organ, is particularly susceptible to IRI which may occur during episodes of hypotension, vascular surgery, sepsis, cardiac events or during kidney retrieval for transplantation (10). Kidney IRI causes inflammation (11, 12), immune system activation (13–15), microvascular dysfunction (16–19) and fibrosis (20, 21). IRI has both immediate and early clinical consequences (acute kidney failure) (22, 23) and contributes to progressive long term fibrosis and chronic kidney disease (CKD) (24). Kidney IRI causes significant patient morbidity and mortality, contributing to increasing healthcare costs (25–31). One of the hallmarks of kidney IRI is acute tubular necrosis, which occurs predominantly in the proximal tubules (9).

Recent studies have established that necrosis can occur in a regulated manner via several pathways including necroptosis (32), ferroptosis (33), pyroptosis (34, 35), mitochondrial permeability transition (MPT)-driven necrosis (36), and parthanatos (37, 38). In contrast to apoptosis, these forms of regulated necrosis lead to cell membrane rupture and release of damage-associated molecular patterns (DAMPs), resulting in inflammation and immune activation [reviewed in (39)]. Necroptosis is a caspase-independent regulated necrosis pathway triggered by a range of extracellular stimuli, including death receptor ligands (e.g. TNF) or pathogen patterns (e.g. LPS) binding to their respective cell surface receptors, or intracellular stimuli such as viral nucleic acids binding to the intracellular receptor ZBP1 (reviewed in (40, 41). In scenarios where the pro-NF-κB activity of the cIAP E3 ubiquitin ligase family and the pro-apoptotic function of Caspase-8 protease are diminished, the intracellular kinases Receptor Interacting Protein Kinase 1 (RIPK1) and Receptor Interacting Protein Kinase 3 (RIPK3) assemble into a high molecular weight intracellular platform termed the necrosome via their RIP homology interaction motifs (RHIMs) (42, 43). Recruitment of subcomplexes comprising RIPK3 and the terminal effector in the pathway – the Mixed lineage kinase domain-like (MLKL) pseudokinase – from the cytosol to the necrosome prompts RIPK3-mediated phosphorylation of the MLKL pseudokinase domain at T357/S358 in human MLKL and S345 in mouse MLKL (44–46). Phosphorylation is the critical step in MLKL activation, inducing MLKL to undergo a conformational change, disengage from the necrosome, and assemble into pro-necroptotic oligomers (47–50). MLKL oligomers are trafficked from the necrosome to the plasma membrane, where they accumulate in hotspots that perturb the membrane to kill cells once a phospho-MLKL (pMLKL) threshold is exceeded (50). Accordingly, phosphorylation of MLKL has become synonymous with necroptosis pathway and MLKL activation, and is considered a hallmark of necroptosis pathway activation. Many of the inflammatory molecules released during necroptosis promote further necroptotic cell death and inflammation (51), creating an auto-amplification loop termed necroinflammation (52).

Necroptosis is believed to play an important role in the pathogenesis of kidney IRI (reviewed in (39) (53). Studies by Linkermann et al. (54) and Newton et al. (55) showed lower mortality of *Ripk3*-deficient mice compared to wild-type (WT) mice. In the absence of mouse-specific inhibitors of MLKL, the executioner protein of necroptosis, *in vivo* data for its role come from studies of necroptosis-deficient *Mlkl* knockout (*Mlkl*-ko) mice (55, 56). Müller et al. used a bilateral renal pedicle clamping model of IRI to demonstrate improved kidney function in *Mlkl*-ko mice compared with wild-type (WT) mice at 48 and 72 hrs post-reperfusion, but not at the earlier timepoints of 6, 12, and 24 hrs (56). Although there was a time-dependent increase in total MLKL protein expression in WT kidneys commencing at 12 hrs post-reperfusion, the authors were unable to detect pMLKL by immunostaining or Western blotting (56). This inability to reliably detect pMLKL in mouse kidney sections has been a major hindrance to understanding the dynamics of necroptosis in kidney IRI. To address this problem, we developed a reproducible method to stain pMLKL in formalin-fixed kidney tissue sections and used it to track the temporal and spatial activation of necroptosis in the kidneys of WT mice subjected to unilateral IRI. Mice were analyzed at different timepoints post-reperfusion (0, 3, 12, 24, 48, 72 hrs, and 4 weeks). The pMLKL staining pattern at each timepoint was correlated with kidney function, tubular injury, and the expression of a range of genes relevant to kidney injury, inflammation, and necroptosis. Finally, we compared *Mlkl*-ko mice with WT littermate controls at each timepoint to assess the degree and mechanism of protection from IRI afforded by deletion of MLKL.

## 2 Materials and methods

### 2.1 Animal and ethical statement

*Mlkl*-ko mice on a C57BL/6J background (45) and WT littermate controls were generated by heterozygous matings and screened by PCR of tail tip genomic DNA by the Walter and Eliza Hall Institute for Medical Research (WEHI) (45). Mice were housed in microisolator cages in a pathogen-free facility with a 12-hour light-dark cycle under standard conditions of temperature and humidity and fed commercial mouse chow diet with free access to drinking water. All animal experiments were conducted in compliance with the Australian Code of Practice for the Care and Use of Laboratory Animals for Scientific Purposes (Eighth edition, 2013) and the Prevention of Cruelty to Animals Act 1986, Victoria, Australia. All experiments were carried out with the approval of the Animal Ethics Committee of St. Vincent’s Hospital Melbourne.

### 2.2 Kidney ischemia-reperfusion injury model

10–12-week-old male mice were anaesthetised by intraperitoneal injection of ketamine (100 mg/kg) and xylazine (15 mg/kg), followed by right nephrectomy prior to left renal pedicle clamping for 18 min using a microvascular clamp (Roboz, Rockville, MD). After removal of the clamp, the kidney was assessed for even reperfusion prior to abdominal wound closure. Mice received 200 µL warm normal saline (37°C) i.p. post-operatively, and core body temperature was maintained at 35.5 - 36.5°C throughout. Sham mice had a right nephrectomy but the left renal pedicle was not clamped. Separate cohorts of mice were euthanised by anesthetic overdose (ketamine/xylazine) and exsanguination immediately following injury (baseline), at 3, 12, 24, 48, 72 hours or 4 weeks after reperfusion. Blood and kidney samples were obtained to assess kidney function, kidney injury, inflammation, and necroptosis. Group sizes varied depending on availability of homozygous *Mlkl-*ko and WT littermates during experimental procedures. Specific group sizes are specified in all figure legends.

### 2.3 Analysis of kidney function

Serum creatinine was measured using a kinetic colorimetric assay on a COBAS Integra 400 Plus analyser (Roche, Castle Hill, NSW, Australia).

### 2.4 Assessment of tubular injury

Kidney tissue blocks were fixed overnight in 10% formalin and embedded in paraffin wax. 3 µm sections were cut and de-waxed prior to Periodic Acid Schiff (PAS) staining (57). Each section was divided into 12 regions. A representative area of each region was viewed under 400X magnification, with a focus on the corticomedullary junction. A score was derived by calculating the number of damaged proximal tubules as a percentage of total proximal tubules manually counted in each area (58). Markers of a damaged tubule included tubular atrophy, tubular dilatation, tubular cast formation, vacuolization, and tubular cell degeneration with loss of brush border or thickening of tubular basement membranes. An average of the 12 scores obtained per section was calculated. Scoring was performed under blinded conditions by personnel trained by an experienced veterinarian pathologist.

### 2.5 Immunohistochemical analysis

3 µm paraffin sections were de-waxed prior to antigen retrieval in a citrate buffer (pH 6.0) using a pressure cooker at 125°C for 90 s (pMLKL staining) or 180 s (cC3 staining), and left to cool to room temperature. Slides were then washed on a shaker prior to incubation in 10 μL 3% H_2_O_2_ v/v in double-distilled water for 5 min and blocked with 50 μL 10% swine serum for 1 hr at room temperature. Sections were then incubated with rabbit monoclonal anti-phospho-MLKL (pSer345) (ab196436, 1:100, Abcam, Melbourne, Australia) or rabbit monoclonal anti-human/mouse cleaved Caspase-3 (cC3) (Asp175) (IC835G, 1:200, RD Systems, Noble Park, Australia) in 2% swine serum at 4°C overnight. After incubation with DAKO anti-rabbit IgG HRP secondary antibody (Envision+ System, K4003, 1:1, Dako, Santa Clara, USA) for 1 hr, sections were developed using DAB and counterstained with hematoxylin. pMLKL- or cC3-stained sections were scanned using an Aperio ScanScope (Leica Biosystems) to generate a digitized image of the whole section. 12 representative areas of the cortex were analysed (4 upper pole, 4 mid pole and 4 lower pole) at X400 magnification. A score was manually calculated by counting the number of tubules with pMLKL or cC3 staining as a percentage of the total number of tubules in the section.

### 2.6 Reverse transcription-quantitative PCR

Harvested kidneys were stored in RNA Later^®^ at 4°C for 24-48 h, followed by storage at -80°C until processing. Total RNA was extracted using the ReliaPrep ™ RNA Tissue Miniprep system (Promega Australia, Alexandria, NSW, Australia) according to manufacturer’s instructions. RNA concentration and quality were measured using a Fluorostar Omega multimode microplate reader (BMG Labtech, Mornington, Australia). First-strand complementary DNA (cDNA) was generated in a reaction volume of 22 µL containing 1 µg oligo (dT), 1 µg random hexamers (Invitrogen, Carlsbad, CA), 12 µg of RNA and sterile Milli-Q H_2_O. The reaction was incubated for 10 min at 70°C. Following this, a 28 µL mix comprising 2.5 µL 10 mM dNTPs, 1 µL SuperScript III recombinant reverse transcriptase, 1 µL RNaseOUT recombinant ribonuclease inhibitors, 2.5 µL 0.1 M DTT, 10 µL 5 x first strand buffer (Invitrogen, Carlsbad, USA) and 11 µL Milli-Q H_2_O was added to the first reaction. Reverse transcription was performed at 42°C for 1 hr and 70°C for 10 min. The cDNA was stored at -20°C. Quantitative real-time PCR was performed using the TaqMan Universal PCR Master Mix system and TaqMan Gene Expression Assays for Kidney injury molecule 1 (*Kim1*) (Mm00506686_m1), Neutrophil gelatinase-associated lipocalin (*Ngal*) (Mm01324470_m1), Tumour necrosis factor alpha (*Tnfa*) (Mm00443258_m1), Interleukin 1 beta (*Il1b*) (Mm00434228_m1), Interleukin 33 (*Il33*) (Mm00505403_m1), Interleukin 6 (*Il6*) (Mm00446190_m1), Macrophage inflammatory protein-2 (*Mip2*) (Mm00436450_m1), Receptor interacting protein kinase 1 (*Ripk1*) (Mm00436354_m1), Receptor interacting protein kinase 3 (*Ripk3*) (Mm00444947_m1), Mixed lineage kinase domain-like protein (*Mlkl*) (Mm01244222_m1), Toll-like receptor 2 (*Tlr2*) (Mm00442346_m1), Toll-like receptor 4 (*Tlt4*) (Mm00445273_m1), and Glyceraldehyde 3-phosphate dehydrogenase (*Gapdh*) (Mm99999915_g1) (Applied Biosystems, Carlsbad, CA), using a 7400Fast Real-Time PCR System (Applied Biosystems).

### 2.7 Statistical analysis

Descriptive statistics were used to summarise the kinetic changes at different time points after reperfusion. Results were expressed as mean ± SEM unless otherwise indicated. A Kruskal-Wallis test and Dunn multiple comparisons test were used when comparing > 2 groups. Comparisons between 2 groups were performed using a Mann-Whitney U-test. Statistical analyses were performed using GraphPad Prism version 9.3 (GraphPad, San Diego, CA) with p<0.05 considered significant.

## 3 Results

### 3.1 Kinetics of kidney injury and pro-inflammatory gene expression in WT mice following IR

**Figure 1:**
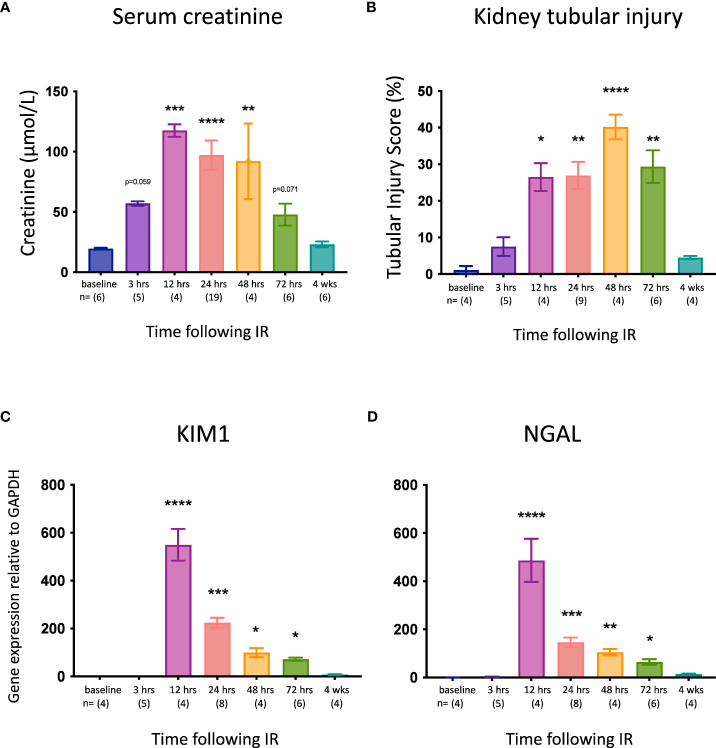
Time course of kidney injury after IR in WT mice. **(A)**: serum creatinine; **(B)**: tubular injury score; **(C)**: mRNA expression of *Kim1*; **(D)**: mRNA expression of *Ngal*. Kruskal-Wallis test with Dunn multiple comparisons test with * p<0.05, ** p<0.01, *** p<0.001, **** p<0.0001 compared to baseline control. Data presented as mean ± SEM.

C57BL/6J WT mice were subjected to right nephrectomy and clamping of the left renal pedicle for 18 min, and cohorts were analyzed at 7 timepoints post-reperfusion (0, 3, 12, 24, 48, 72 hrs, and 4 weeks). Kidney injury was assessed by measuring kidney function (serum creatinine), tubular injury (semi-quantitative morphological analysis), and mRNA expression of the genes for the kidney injury markers KIM-1 and NGAL. Serum creatinine (**Figure 1A**) and tubular injury (**Figure 1B**; **Supplementary Figure 2**) were elevated at 3 hrs, although these increases did not reach statistical significance and there was no change in expression of *Kim1* (**Figure 1C**) or *Ngal* (**Figure 1D**) at this early timepoint. By 12 hrs, creatinine levels and tubular injury were significantly increased, and expression of both *Kim1* and *Ngal* was massively upregulated. All parameters remained elevated at 24, 48 and 72 hrs before returning to baseline by 4 weeks.

**Figure 2:**
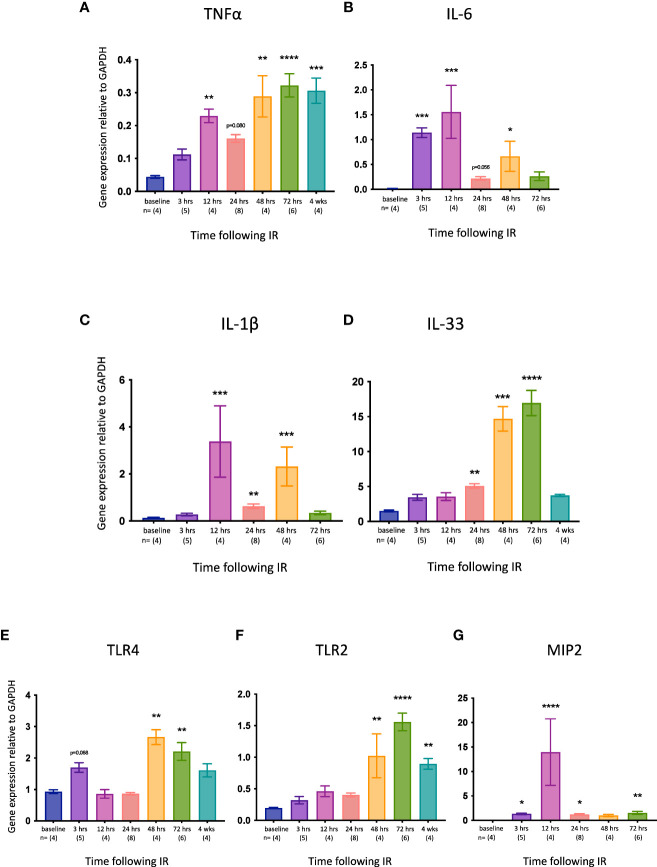
Time course of pro-inflammatory gene expression in the left kidney after IR in WT mice. **(A)**: *Tnfa*; **(B)**: *Il6*; **(C)**: *Il1b*; **(D)**: *Il33*; **(E)**: *Tlr4*; **(F)**: *Tlr2*. **(G)**: *Mip2*. Kruskal-Wallis test with Dunn multiple comparisons test with * p<0.05, ** p<0.01, *** p<0.001, **** p<0.0001 compared to baseline control. Data presented as mean ± SEM.

We then examined the expression of several pro-inflammatory genes relevant to necroptosis and/or kidney IRI. TNFα is the best characterized activator of necroptosis (53) and has been described as a necroptosis-associated alarmin (59). *Tnfa* expression was significantly increased at 12, 48 and 72 hrs, and remained high at 4 weeks (**Figure 2A**). Interleukin-6 (IL-6) is an acute inflammatory cytokine that is upregulated in necrotic cells but not in apoptotic cells (60). *Il6* expression was elevated earlier than *Tnfa* (3 hrs) and returned to baseline levels by 72 hrs (**Figure 2B**). IL-1β and IL-33 are released by necroptotic cells (61, 62). *Il1b* expression was upregulated at 12, 24 and 48 hrs (**Figure 2C**), whereas *Il33* was increased later, from 24 hrs (**Figure 2D**). Signalling via Toll-like receptor 4 (TLR4) can activate necroptosis, whereas TLR2 has not been shown to play a role in necroptotic cell death (53). Both *Tlr4* (**Figure 2E**) and *Tlr2* (**Figure 2F**) were upregulated relatively late (48 and 72 hrs, respectively), with *Tlr2* expression remaining high at 4 weeks. Macrophage inflammatory protein-2 (MIP-2) is a neutrophil chemoattractant/activator that is upregulated in kidney IRI (63). *Mip2* expression was significantly increased at all timepoints except 48 hrs, peaking at 12 hrs (**Figure 2G**).

### 3.2 Kinetics of necroptotic pathway gene expression in WT mice following IR

**Figure 3:**
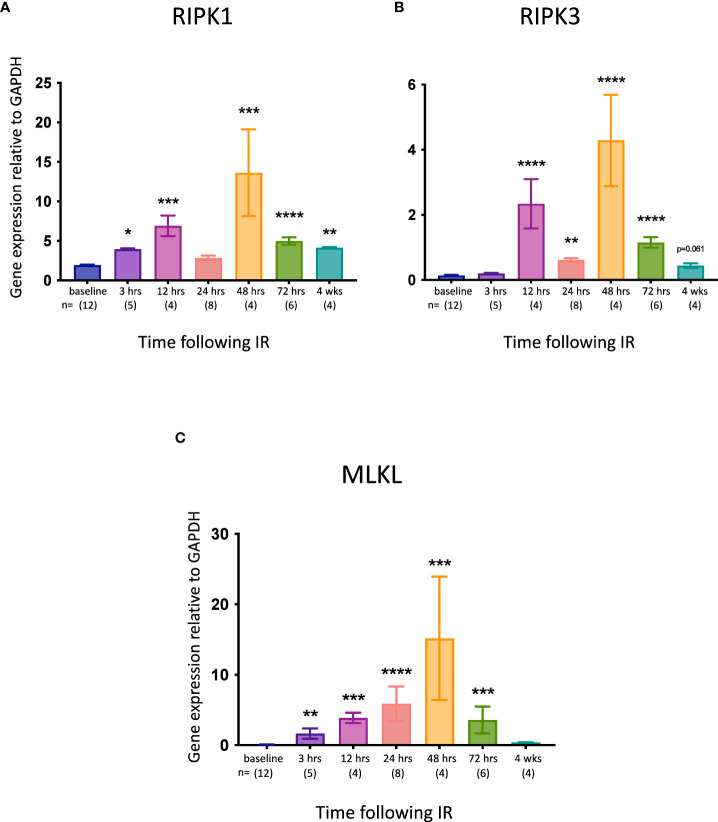
Time course of necroptotic pathway gene expression in the left kidney after IR in WT mice. **(A)**: *Ripk1*; **(B)**: *Ripk3*; **(C)**: *Mlkl*. Kruskal-Wallis test with Dunn multiple comparisons test with * p<0.05, ** p<0.01, *** p<0.001, **** p<0.0001 compared to baseline control. Data presented as mean ± SEM.

Although elevated expression of the necroptotic pathway genes *Ripk1*, *Ripk3* and *Mlkl* does not necessarily equate with increased necroptosis *in vivo* (53), it may indicate an increased propensity for this form of cell death. We therefore measured IRI-associated changes in the expression of these genes in the kidney at different times post-reperfusion. All 3 genes were significantly upregulated at most timepoints, with the highest expression observed at 48 hrs (**Figure 3**).

### 3.3 Temporal and spatial pattern of pMLKL staining in kidneys of WT mice following IR

**Figure 4:**
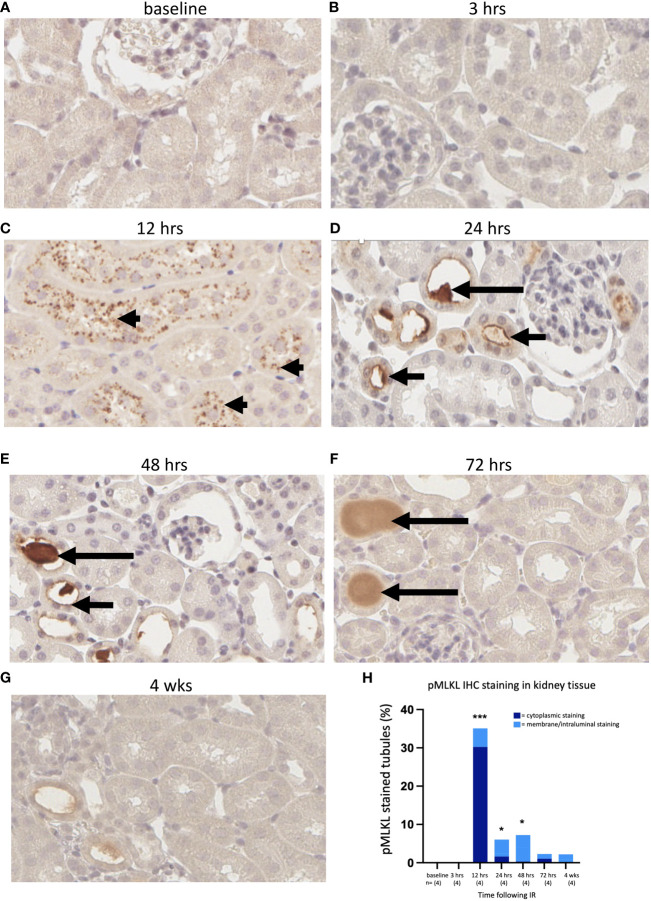
Time course of pMLKL staining in the left kidney after IR in WT mice. Representative sections from 0 hrs **(A)**, 3 hrs **(B)**, 12 hrs **(C)**, 24 hrs **(D)**, 48 hrs **(E)**, 72 hrs **(F)**, and 4 weeks **(G)** post-reperfusion. **(H)**: percentage of proximal tubules staining positive for pMLKL, stratified according to cellular location (dark blue = cytoplasmic; light blue = membrane/intraluminal). Examples of cytoplasmic, membrane and proteinaceous cast staining are indicated by arrowheads and short and long arrows, respectively. Kruskal-Wallis test with Dunn multiple comparisons test with * p<0.05, *** p<0.001 compared to baseline control. Image magnification x 400.

Sections were cut from formalin-fixed left kidney tissue of WT mice from each cohort and stained for pMLKL using a rabbit monoclonal antibody [ab196436, EPR9515 (2)] This antibody has previously been used to detect pMLKL in methanol-fixed mouse dermal fibroblast cell lines (64) and in formalin-fixed mouse cecal tissue (65). Reproducible staining was achieved after optimization of the antigen retrieval step based initially on a method described by He et al., 2021 (65). *Mlkl*-ko mice subjected to IRI were used as a control to validate the antibody; *Mlkl*-ko kidneys showing substantial morphological injury were negative for pMLKL staining at each timepoint (**Supplementary Figure 3**). pMLKL was not detected in WT/IRI kidneys at 0 or 3 hrs post-reperfusion (**Figures 4A, B**). At 12 hrs, numerous tubules containing cells displaying dense punctate cytoplasmic staining were observed (**Figure 4C**). Interestingly, the tubules at this timepoint were either completely positive or completely negative. At 24 hrs, the pattern of staining had changed from cytoplasmic to predominantly membrane/intraluminal i.e., on the apical surface of tubular epithelial cells (**Figure 4D**). At 48 hrs, strong staining of proteinaceous casts within the tubules was observed, along with patchy intraluminal staining (**Figure 4E**). At 72 hrs (**Figure 4F**) and 4 weeks (**Figure 4G**), staining was weaker and mainly restricted to casts.

We performed a semi-quantitative analysis by counting the number of pMLKL-positive tubules as a percentage of the total number of tubules. The degree of positivity was highest at 12 hrs and remained significantly elevated at 24 and 48 hrs (**Figure 4H**). The data were further stratified into cytoplasmic and intraluminal staining, likely representing the activation and execution phases of necroptosis, respectively. On this basis, maximum activation of necroptosis was observed at 12 hrs, while the terminal stage of necroptotic cell death was relatively constant at 12, 24 and 48 hrs, and returned to close to baseline by 72 hrs (**Figure 4H**).

### 3.4 Effect of MLKL deletion on kidney IRI

**Figure 5:**
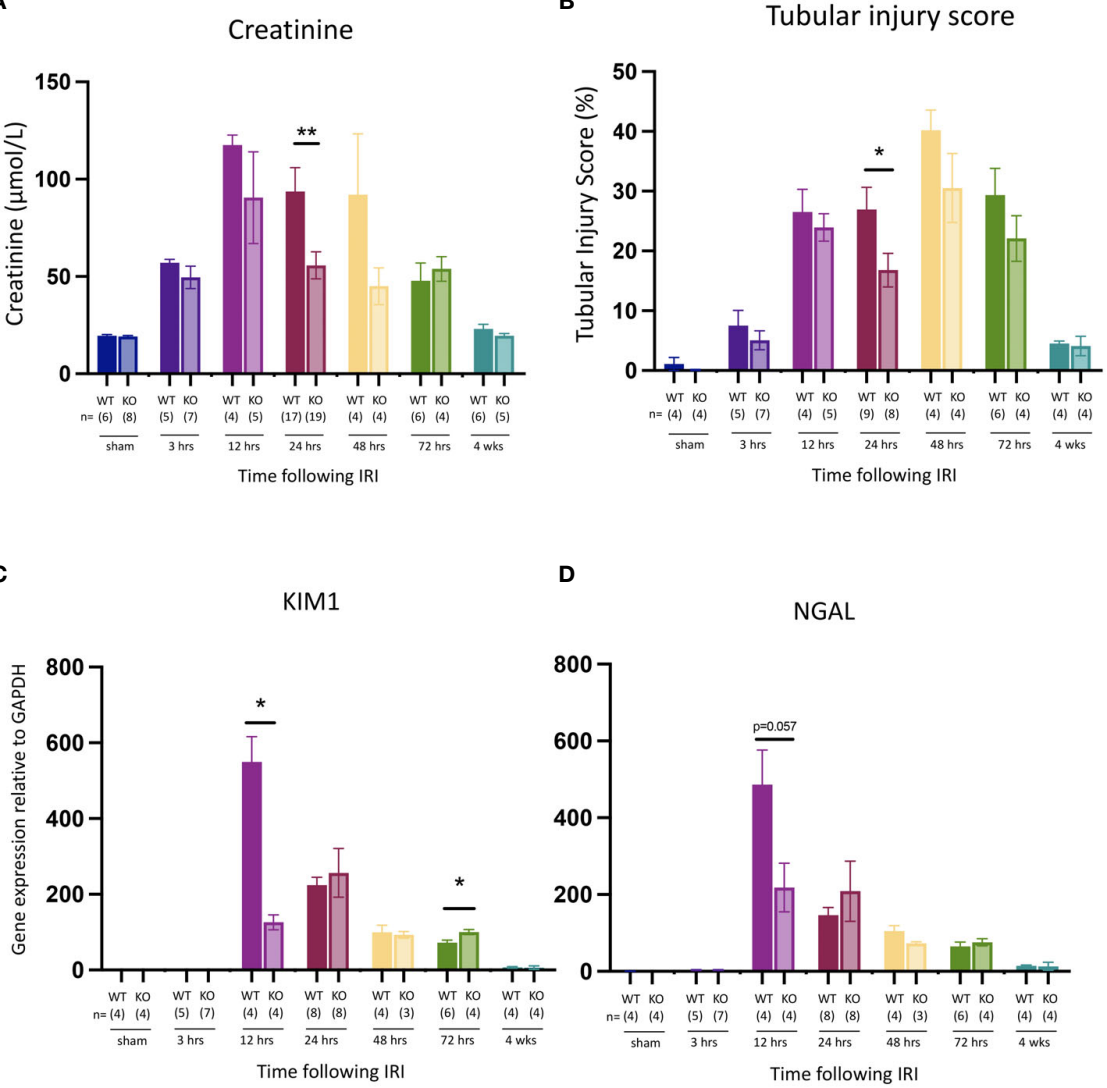
Comparison of kidney injury time course after IR in *Mlkl*-ko and WT mice. **(A)**: serum creatinine; **(B)**: tubular injury score; **(C)**: mRNA expression of *Kim1*; **(D)**: mRNA expression of *Ngal*. Mann Whitney U test comparing with *Mlkl*-ko and WT mice at each timepoint; * p<0.05, ** p<0.01. Data presented as mean ± SEM.

Compared to WT mice, *Mlkl*-ko mice showed reduced kidney injury at 24 hrs post-reperfusion, evidenced by significant decreases in serum creatinine (**Figure 5A**) and tubular injury score (**Figure 5B**). Kidney function and injury were not significantly different at the other timepoints. Expression of *Kim1* (**Figure 5C**) and *Ngal* (**Figure 5D**) were reduced at the earlier timepoint of 12 hrs, although only the former was statistically significant. Expression of the pro-inflammatory cytokine genes *Tnfa*, *Il1b* and *Mip2* was also significantly lower at 12 hrs in *Mlkl*-ko mice compared to WT mice, whereas expression of *Il6*, *Il33*, *Tlr2* and *Tlr4*, and of the necroptosis pathway genes *Ripk1* and *Ripk3*, was generally similar in *Mlkl*-ko and WT mice at all timepoints (**Supplementary Figure 1**). As expected, *Mlkl* expression and pMLKL staining were undetectable in kidneys from *Mlkl*-ko mice (data not shown).

**Figure 6:**
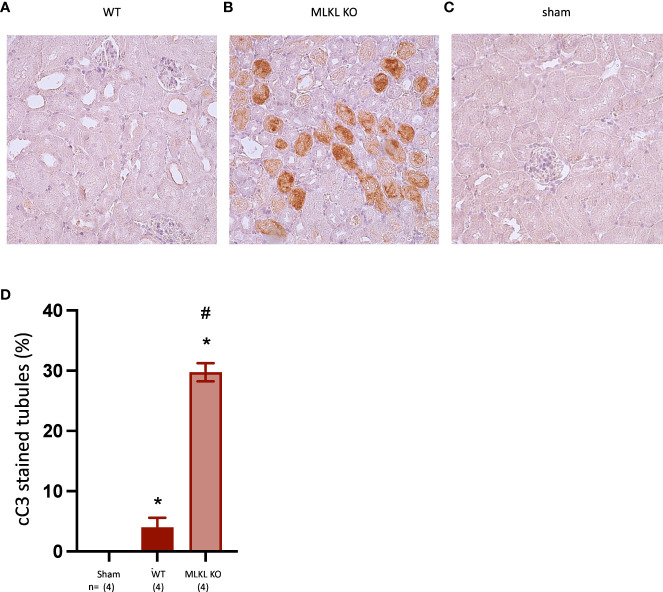
Comparison of cleaved Caspase-3 staining in WT and *Mlkl*-ko mice at 24 hrs post-reperfusion. **(A)**: WT; **(B)**: *Mlkl*-ko; **(C)**: sham; **(D)**: semi-quantitative analysis. Mann Whitney U test with * p<0.05 compared to sham procedure and # p<0.05 compared to WT mice. Data presented as mean ± SEM. Image magnification x 200.

To investigate the mode of IRI-associated cell death in a necroptosis-deficient setting, kidney sections from the 24 hr timepoint were stained for cleaved Caspase-3 (cC3) as a marker of apoptosis. While WT kidneys showed a significant but modest increase in apoptotic tubules compared to sham kidneys, *Mlkl*-ko kidneys showed a major increase (**Figure 6**), suggesting a greater role for apoptosis in this model when necroptosis is inactivated. The pattern of cC3 staining in *Mlkl*-ko tubules at 24 hrs was like that of pMLKL staining in WT tubules at 12 hrs (**Figure 4C**) i.e., tubules were either completely positive or negative.

## 4 Discussion

Although several studies employing mouse models have implicated necroptosis in the pathogenesis of kidney IRI (54, 55, 66, 67), this is the first study to definitively demonstrate activation of the terminal stage of the necroptotic pathway (i.e., phosphorylation of MLKL) in this setting and to explore the dynamics of the process. We performed a time course analysis in a mouse model of unilateral warm IRI to detail the kinetics of kidney injury, inflammation and necroptosis. Our data suggest that the critical phase lies within 3-12 hrs following reperfusion. During this period, there was major activation of necroptosis as evident by strong pMLKL staining within proximal tubular cells (PTCs) at 12 hrs. Some of the PTCs exhibited pMLKL staining at the cell surface, suggesting progression to the pre-lytic/lytic phase of necroptosis. At the same time, there was upregulated expression of the necroptosis pathway genes *Ripk1*, *Ripk3* and *Mlkl* and the pro-inflammatory cytokine genes *Tnfa*, *Il6*, *Il1b* and *Mip2*. This, together with activation of the NLRP3 inflammasome by pMLKL (68), would be expected to create favourable conditions for further necroptosis and inflammation via DAMP release, driving the cycle of necroinflammation.

Of interest was the pattern of pMLKL staining. Epithelial cell injury associated with kidney IR is most apparent in the proximal tubules (1), as PTCs have low anaerobic glycolytic capacity and are located in areas with low partial pressure of oxygen such as the outer medulla and inner cortex (9). Consistent with this, we observed that tubular injury was focused in the proximal tubules in the cortico-medullary region. Necroptosis, as indicated by pMLKL staining, was also restricted to PTCs. This is in contrast to the findings of a recent single-cell analysis suggesting that necroptosis in kidney IRI occurs mainly in non-PTC cell types, with secondary effects on PTCs (69). Furthermore, injured tubules were clustered together, and necroptosis appeared to be activated in all cells within affected tubules. This suggests either a location-dependent effect, or communication between adjacent cells undergoing necroptosis resulting in the spread of necroptosis from cell to cell within affected tubules. pMLKL staining from 24 hrs appeared polarised, with staining of the apical but not basal surface of PTCs. The precise mechanism by which pMLKL clustering at the apical surface may promote cellular communication within the lumen of affected tubules remains to be further explored, with roles for pMLKL in promoting plasma membrane remodelling at intercellular junctions (50) and generation of extracellular vesicles (70, 71) previously proposed.

In the absence of necroptosis (i.e., in *Mlkl*-ko mice), early inflammation and kidney injury were reduced, providing further evidence that necroptosis and the associated inflammation play a role in kidney damage following IR. The absence of pMLKL staining at 3 hrs in WT mice indicated that the initial injury did not involve necroptosis. It is therefore unsurprising that there was no difference in serum creatinine [which is a delayed marker of kidney injury (72)] between WT and *Mlkl*-ko mice at 3 hrs. The inability of *Mlkl*-ko mice to activate necroptosis in the critical 3-12 hr phase is likely to account for the subsequent reduction in kidney inflammation at 12 hrs, and lower serum creatinine and tubular injury scores at 24 hrs. Interestingly, *Mlkl*-ko mice were protected from kidney IRI at 24 hrs but not at 48 or 72 hrs. This contrasts with an earlier study where protection was noted at 48 and 72 hrs but not at 24 hrs (56). Model differences, including the type (unilateral versus bilateral) and degree of ischemia (18 min versus 35 min), anesthesia (ketamine/xylazine versus the more IRI-protective isoflurane), and WT controls (littermates versus non-littermates), may account for this discrepancy.

While we have further defined the role of necroptosis in kidney IRI, the stimulus that triggers necroptosis in this setting remains unclear. Engagement of death receptors such as TNF receptor 1, activation of TLRs, interferon signalling, and intracellular stimuli in response to viruses, are all able to initiate necroptosis (73). A recent *in vitro* study using a genetically manipulated myeloid cell system confirmed TNF as an alarmin molecule released following necroptotic cell death to drive further inflammation (59). We suggest that TNF released by inflammatory cells triggers necroptosis during the 3-12 hours post-reperfusion, while TNF and DAMP release following necroptotic cell death stimulate subsequent waves of necroptosis. We showed upregulation of *Tnfa* gene expression in WT mice, which was reduced in *Mlkl*-ko mice, leading us to hypothesise that TNF activates necroptosis and drives ongoing necroinflammation in kidney IRI.

The concept that one regulated cell death pathway can compensate for another in acute kidney injury has previously been proposed for necroptosis and ferroptosis, although treatment of *Mlkl*-ko mice with a ferroptosis inhibitor did not provide further protection from kidney IRI (56). In our model, apoptosis appeared to become the dominant cell death pathway in the absence of necroptosis, suggesting a relationship between necroptosis and apoptosis in kidney IRI. However, a limitation of our study was that it focused on the role of necroptosis without investigating the contribution of other regulated necrosis pathways, notably ferroptosis.

Our study is the first to describe the dynamics of necroptosis and necroinflammation in a mouse model of kidney IRI. Our results suggest that treatments targeting the key necroptosis component, MLKL, may reduce kidney injury following IR, although it should be noted that no specific inhibitors of mouse MLKL are currently available. In addition, our results suggest that such treatments may be effective even if given 3 hrs after reperfusion. This is advantageous in a clinical setting, as in many instances IRI is unpredictable and cannot be treated prophylactically. Finally, combination therapy with inhibitors of MLKL and apoptosis may provide additive protection, with the ultimate aim of preventing acute and chronic kidney disease following kidney IR.

## Data availability statement

The raw data supporting the conclusions of this article will be made available by the authors, without undue reservation.

## Ethics statement

The animal study was approved by St Vincent’s Hospital Melbourne Animal Ethics Committee. The study was conducted in accordance with the local legislation and institutional requirements.

## Author contributions

AP, JM, FI and PC designed the experimental studies. AP, AB, JM, NF, ES conducted the experimental studies. AB, JM and AP performed the IRI surgery. AP, AB, ES and JM analysed the results. AP and PC drafted the manuscript. All authors contributed to the article and approved the submitted version.

## Supplementary material

Supplementary Figure 1 Comparison of pro-inflammatory and necroptotic gene expression after IR in *Mlkl*-ko and WT mice. **(A)**: *Tnfa*; **(B)**: *Il6*; **(C)**: *Il1b*; **(D)**: *Il33*; **(E)**: *Tlr4*; **(F)**: *Tlr2*; **(G)**: *Mip2*; **(H)**: *Ripk1*; **(I)**: *Ripk3*. Mann Whitney U test comparing with *Mlkl*-ko and WT mice at each timepoint; * p<0.05. Data presented as mean ± SEM.

Supplementary Figure 2 Representative PAS stained kidney tissue at baseline, 3 hrs, 12 hrs, 24 hrs, 48 hrs, 72 hrs and 4 weeks following IRI. Image magnification x 40.

Supplementary Figure 3 Representative pMLKL stained kidney tissue of *Mlkl*-ko mice taken at baseline, 3 hrs, 12 hrs, 24 hrs, 48 hrs, 72 hrs and 4 weeks following IRI. Image magnification x 40.
