# Supplementary figures and images for: Dynamics of necroptosis in kidney ischemia-reperfusion injury
Source: Front Immunol. 2023 Nov 2;14:1251452. doi: 10.3389/fimmu.2023.1251452 (PMC10652410; doi:10.3389/fimmu.2023.1251452)

Suppl Fig 1

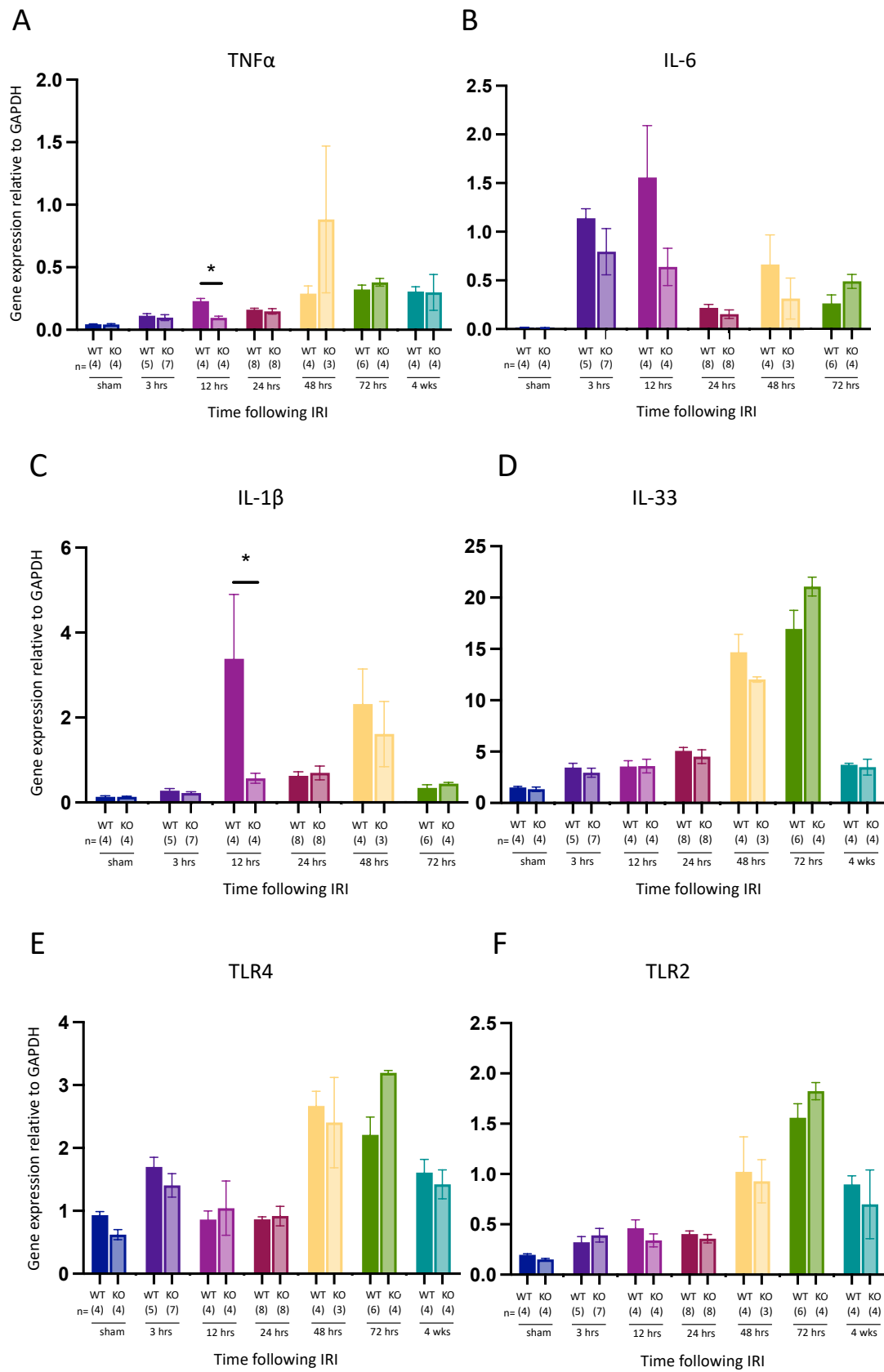

G

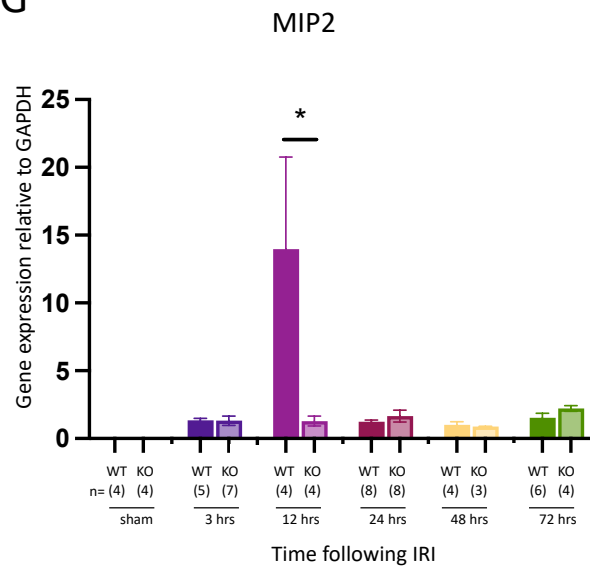

H

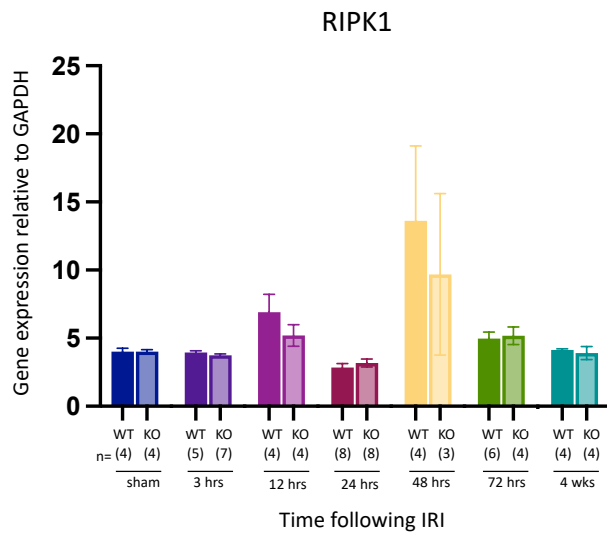

I

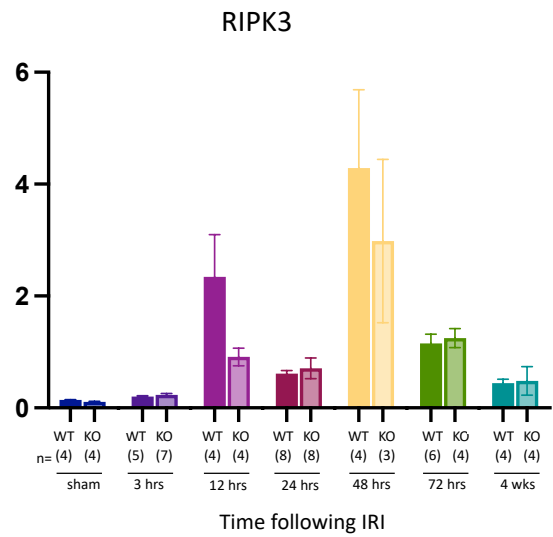

Supplement: Supplementary Figure 1 — Comparison of pro-inflammatory and necroptotic gene expression after IR in Mlkl-ko and WT mice. (A): Tnfa; (B): Il6; (C): Il1b; (D): Il33; (E): Tlr4; (F): Tlr2; (G): Mip2; (H): Ripk1; (I): Ripk3. Mann Whitney U test comparing with Mlkl-ko and WT mice at each timepoint; * p<0.05. Data presented as mean ± SEM. [file Image_1.pdf]

Suppl Fig 2

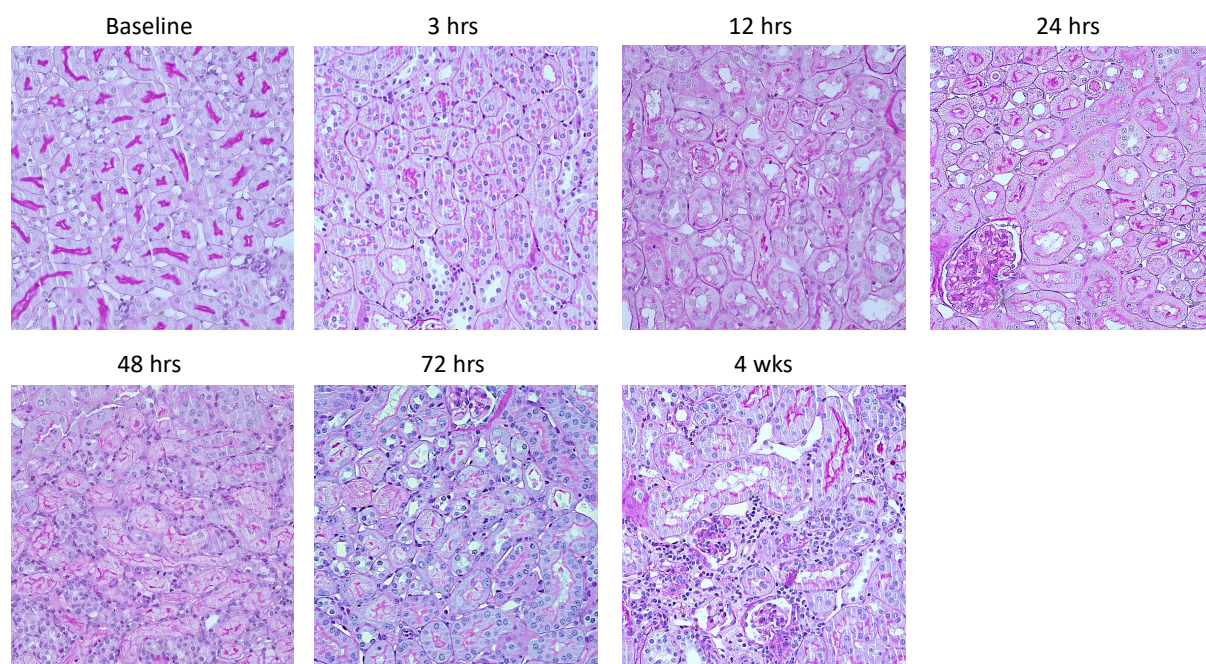

Supplement: Supplementary Figure 2 — Representative PAS stained kidney tissue at baseline, 3 hrs, 12 hrs, 24 hrs, 48 hrs, 72 hrs and 4 weeks following IRI. Image magnification x 40. [file Image_2.pdf]

Suppl Fig 3

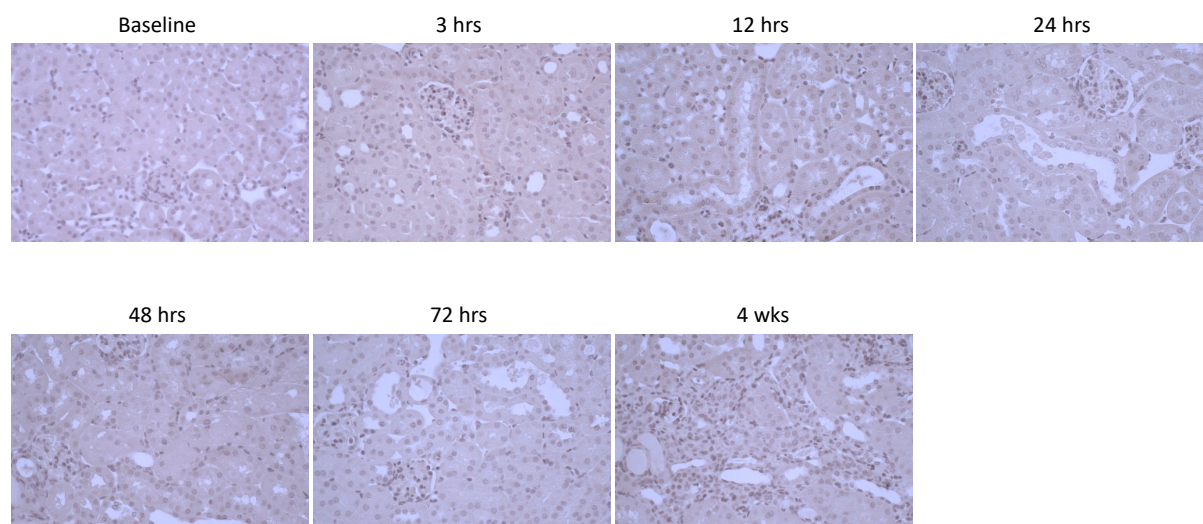

Supplement: Supplementary Figure 3 — Representative pMLKL stained kidney tissue of Mlkl-ko mice taken at baseline, 3 hrs, 12 hrs, 24 hrs, 48 hrs, 72 hrs and 4 weeks following IRI. Image magnification x 40. [file Image_3.pdf]
